# Apomorphine Targets the Pleiotropic Bacterial Regulator Hfq

**DOI:** 10.3390/antibiotics10030257

**Published:** 2021-03-04

**Authors:** Florian Turbant, David Partouche, Omar El Hamoui, Sylvain Trépout, Théa Legoubey, Frank Wien, Véronique Arluison

**Affiliations:** 1 Laboratoire Léon Brillouin LLB, CEA, CNRS UMR12, Université Paris Saclay, CEA Saclay, 91191 Gif-sur-Yvette, France; Florian.Turbant@cea.fr (F.T.); David.partouche2@ext.soprasteria.com (D.P.); thea.legoubey@cea.fr (T.L.); 2 Synchrotron SOLEIL, L’Orme des Merisiers, Saint Aubin BP48, 91192 Gif-sur-Yvette, France; omar.el-hamoui.omar@univ-pau.fr; 3 Institut Curie, Inserm US43, and CNRS UMS2016, 91405 Orsay CEDEX, France; Sylvain.Trepout@curie.fr; 4 UFR Sciences du Vivant, Université de Paris, 75006 Paris CEDEX, France

**Keywords:** apomorphine, bacterial amyloid, functional amyloid, protein fibrils, protein fibrillation inhibition, Hfq, bacterial adaptation, antibacterial compound

## Abstract

Hfq is a bacterial regulator with key roles in gene expression. The protein notably regulates translation efficiency and RNA decay in Gram-negative bacteria, thanks to its binding to small regulatory noncoding RNAs. This property is of primary importance for bacterial adaptation and survival in hosts. Small RNAs and Hfq are, for instance, involved in the response to antibiotics. Previous work has shown that the *E. coli* Hfq C-terminal region (Hfq-CTR) self-assembles into an amyloid structure. It was also demonstrated that the green tea compound EpiGallo Catechin Gallate (EGCG) binds to Hfq-CTR amyloid fibrils and remodels them into nonamyloid structures. Thus, compounds that target the amyloid region of Hfq may be used as antibacterial agents. Here, we show that another compound that inhibits amyloid formation, apomorphine, may also serve as a new antibacterial. Our results provide an alternative in order to repurpose apomorphine, commonly used in the treatment of Parkinson’s disease, as an antibiotic to block bacterial adaptation to treat infections.

## 1. Introduction

Antibiotic resistance happens when a bacterium fights a compound developed to kill it. This existed before antibiotics were used by humans [1]. Indeed, antibiotic resistance occurs naturally through mutations and natural selection. The antibiotic action can be considered an environmental pressure. Thus, bacteria that have a mutation allowing them to survive in the presence of an antibiotic will be selected from others. This results in the constant emergence of new resistant pathogens that have acquired new resistance mechanisms. The inappropriate use of antibiotics over years has induced an increasing bacterial resistance and the appearance of multidrug-resistant (MDR) bacteria. Indeed, some bacterial strains have acquired resistance to more or less all existing antibiotics, which use four main mechanisms (i.e., inhibition of cell wall, protein or nucleic acid synthesis, or of metabolic pathways such as folate synthesis). This acquisition of resistance may consist in antibiotic modification, destruction or efflux [2]. For the past years, the development of “new” antibiotics consisted primarily of chemical derivatives of previously existing antibiotics. Nevertheless, this strategy resulted in a rapid new resistance acquisition. Identifying new antibiotics based on a novel mechanism of action is therefore of prime importance in the fight against MDR bacteria.

Due to their two membranes, Gram-negative bacteria are particularly resistant to antibiotics. Recent research thus aims at exploring new antibiotics that target proteins involved in Gram-negative bacterial adaptation and help them respond to the changes encountered in their host. Only this adaptation allows the bacteria to survive. To achieve this goal, bacteria operate at two levels: regulation at the transcriptional level [3] or at the post-transcriptional level, which allows a faster response to the changing environment [4]. One of the most promising approaches targeting post-transcriptional regulation consists in blocking noncoding RNA-based (ncRNA) regulation [5]. Many ncRNA from different bacterial species have been identified. They are, on average, hundreds of nucleotides long, hence their name of small RNA (sRNA). In *Escherichia coli*, it is likely that a few hundred sRNAs exist. Many of these sRNA bind mRNAs via imperfect base-pairing, and for this reason a protein called Hfq is usually needed to facilitate RNA’s annealing [6,7,8]. Due to the diversity of their targets, sRNA and Hfq are involved in important bacterial processes, including virulence and pathogenicity [9]. As an example, the acquisition of iron in the infected host is particularly critical, and sRNA with Hfq help to respond to iron starvation [10].

Although Hfq is important in many bacterial pathogens [11], its exact role in drug resistance is still unclear. Recently, its involvement in response to antibiotics was observed in Gram-negative bacteria [12]. Indeed, Hfq mediates drug resistance by regulating the efflux system [13]. For this reason, Hfq has attracted considerable attention, as it could be a promising target for developing new antibiotics against several Gram-negative bacteria: blocking the Hfq function may depress the bacterial adaptive capacity and resistance to antibiotics [13,14]. Structurally, Hfq belongs to the Sm protein family, which is also involved in RNA-related processes such as splicing [15]. Hfq forms a toroidal ring similar to that of Sm proteins [16]. The N-terminal region of Hfq (NTR, 65 amino acids residues) is formed by an antiparallel β-sheet following an amino-terminal α-helix; the NTR bent β-sheets of each monomer interact in order to form the hexameric torus [17]. Many membrane-proteins are regulated by the Hfq-NTR region, including porins involved in antibiotic import and/or efflux [18]. For this reason, attempts have been made to block the Hfq–NTR interaction with sRNA to affect virulence [19]. On the other hand, Hfq comprises a C-terminal region (CTR) of about 40 amino acid residues, located outside of the NTR-ring [20]. No 3D structure is known for this CTR [7]. Nevertheless, the CTR can self-assemble into an amyloid-like structure with repetitive stacked β-strands in vitro and in vivo [21,22]. This CTR region has been shown to play a major role in DNA compaction and to affect the cell membrane [21,23]. We thus hypothesize that interfering with the formation of the Hfq-CTR amyloid structure may have a direct consequence for bacterial survival.

Many amyloid inhibitors have been designed [24]. Historically, these inhibitors have been used to target pathogenic amyloids to treat neurodegenerative diseases. Indeed, the accumulation of amyloids is a characteristic of neurodegenerative disorders such as Alzheimer’s and Parkinson’s diseases. Nevertheless, functional amyloids also exist in nature [25]. Among them, functional amyloids form in bacteria [26]. They can form in either the cytoplasm, as in the case of Hfq, or on the cell surface, for instance with proteins constituting curli [27]. As bacterial amyloids share structural properties with eukaryotic amyloids, inhibitors used against these pathogenic amyloids may thus be effective in altering the bacterial amyloid structure and thus bacterial survival. This has already been proven using Epigallo catechin gallate (EGCG) to target the amyloid region of Hfq or proteins constituting curli [28,29]. Thus, a bacterial amyloid inhibitor against the Hfq-CTR region may provide a valid antimicrobial agent.

Among treatments used in neurodegenerative diseases, apomorphine is often used in the treatment of Parkinson’s disease [30]. Its hydrophobic structure allows it to cross the cell membrane, and it could thus be particularly effective in Gram-negative bacteria. In the past, apomorphine has been tried for different uses, including for reducing anxiety, as a sedative, for craving in alcoholism, to induce vomiting, or to treat erectile dysfunction [31]. Nevertheless, the use of apomorphine was limited due to the hepatic metabolism and side effects. Now, with the progresses in drug delivery, apomorphine is better tolerated and more widely used [31]. In this manuscript, we describe promising preliminary results that may propose apomorphine or its derivatives as new antibiotics.

## 2. Results

### 2.1. Disruption of Hfq CTR Amyloid Fibrils by Apomorphine In Vitro

**Figure 1:**
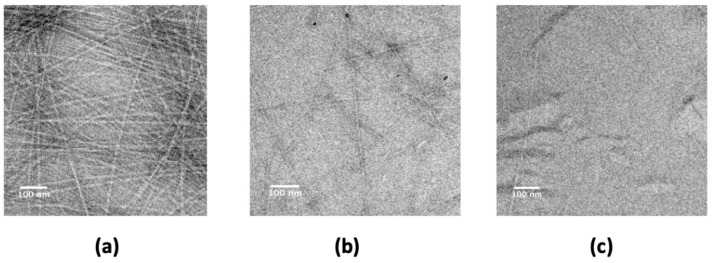
TEM visualization of the effect of apomorphine on CTR fibrils. (**a**) preassembled fibrils of CTR (control). More than 100 fibers can be observed; (**b**) preassembled CTR incubated with 5 mM apomorphine; Incubation time 24 h. Here, only tens of fibers are still present. (**c**) apomorphine was added before the fibrils’ formation, and almost no fibers can be observed in this case. Scale bars, 100 nm.

To investigate the possibility that the Hfq-CTR amyloid can be affected by apomorphine in vitro, we first screened its effect on Hfq-CTR preformed fibrils using transmission electron microscopy (TEM). In order to allow a statistical approach, we used a negative staining procedure and not Cryo Transmission Electron Microscopy (cryo-TEM). Potential amyloid interference was first tested on preformed Hfq-CTR fibrils (amino acid sequence in the Section 4). Fibrils were pre-formed at 20 mg/mL for one month, and then apomorphine (or water as a control) was added for 24 h (Figure 1). As seen by TEM, the addition of apomorphine (5 mM) did not result in the total disappearance of CTR preformed fibrils. Nevertheless, a significantly lower number of fibrils was observed (compare Figure 1b and Figure 1a). Then, we tested the effect of apomorphine on the fibril formation. To this end, apomorphine was added at the beginning of the reaction, before fibers formed. As seen on Figure 1c, the spontaneous self-assembly of Hfq-CTR was drastically affected by the presence of apomorphine: only rare fibers were observed (under the same conditions of incubation as those used in Figure 1a). This means that apomorphine inhibits both the formation of amyloid fibrils, but also that it binds to existing fibrils to disrupt them into small aggregates.

**Figure 2:**
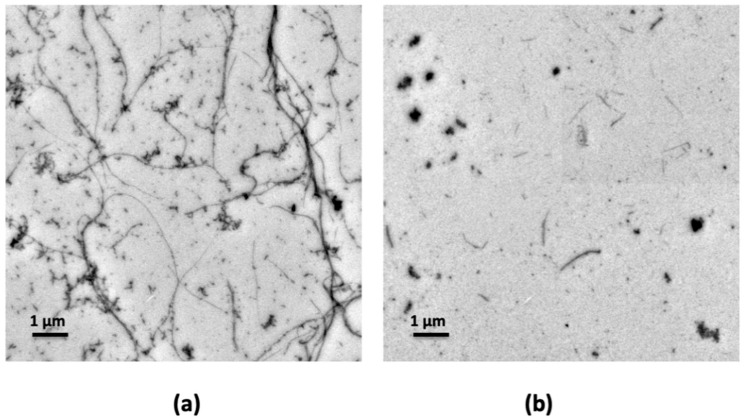
TEM visualization of the effect of apomorphine of Hfq-CTR bound to DNA. (**a**) Hfq-CTR:DNA without apomorphine (control); (**b**) Hfq-CTR:DNA with apomorphine. Only a few pieces of fibrils remain on the grid. Scale bars, 1 µm.

As Hfq-CTR fibrillation is accelerated by DNA [32] and may result in a more stable complex, TEM analyses have also been performed with apomorphine and Hfq-CTR bound to DNA (compare Figure 2b and Figure 2a). As shown, apomorphine does not only disrupt Hfq-CTR preformed fibrils but also disrupts Hfq-CTR fibrils assembled on DNA.

### 2.2. Kinetics of Hfq-CTR Amyloid Disassembly in the Presence of Apomorphine

**Figure 3:**
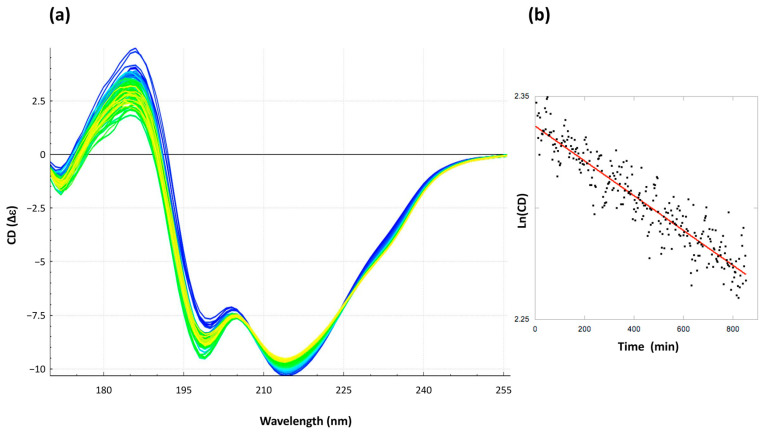
Disassembly of the Hfq-CTR amyloid structure by apomorphine between 0 and 15 h (blue to yellow). (**a**) The decrease in the signal at 215 nm, characteristic of an amyloid structure [32,35], is observed upon the addition of apomorphine. This proves that the amyloid cross-β structure is disrupted by the compound. (**b**) Kinetics of disassembly. Ln of the absolute value of CD at 215 nm vs. time. In our case [Apomorphin] >> [Hfq-CTR_n_] (Hfq-CTR_n_ = Hfq-CTR fibers) and CD_215_ = CD^0^_215._e^−kt^. An apparent dissociation constant k = 7.8 10^−5^ min^−1^ can be measured. CD^0^_215_ is the absolute value of CD at 215 nm and at t = 0 min.

The effect of apomorphine on the Hfq-CTR secondary structure was analyzed. For this, Circular Dichroism (CD) (more precisely, Synchrotron Radiation Circular Dichroism (SRCD)) was used, which allows extending the wavelength range down to 170 nm for the identification and distinction of amyloid signals [33,34]. Aggregation into β-sheets in an amyloidal structure is implied by significant SRCD spectral changes, mainly a negative band at ~215 nm (Figure 3) [32]. The decrease and shift of this band from ~215 nm to ~200 nm observed upon apomorphine addition (Figure 3) suggests that the amyloid cross-β structure is disrupted by the compound, while the same peptide in the absence of apomorphine remains assembled. The Bestsel analysis [33,34], an algorithm that provides an improved β-structure determination from the CD spectrum, indicates that in the presence of apomorphine, antiparallel β-sheets (mainly right-handed twisted, in agreement with a previous report [22]) decrease from 19 to 17% to be converted into a random coil structure.

### 2.3. Apomorphine Affects Bacterial Survival Due to Its Interaction with Hfq CTR

**Figure 4:**
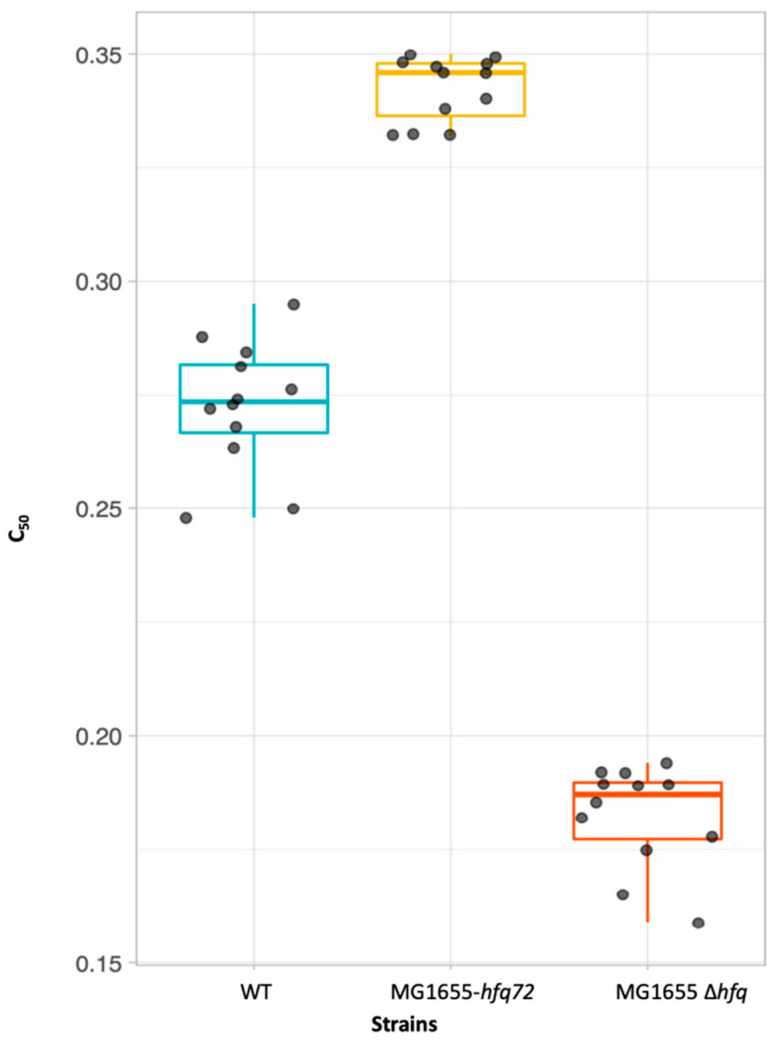
Box plots for C_50_ apomorphine concentrations (in mM) for MG1655 WT, MG1655-∆*hfq* and MG1655-*hfq72* strains. All three strains had different medians (All *p*-values for all pairwise comparisons were smaller than 2 × 10^−5^).

Finally, the effect of apomorphine on bacterial survival was evaluated. The bacterial strains that were used were MG1655 WT, MG1655-∆*hfq* and MG1655-*hfq72* (truncated protein with only the first 72 amino acids) [32]. As described previously in Partouche et al. [28], the concentration of the apomorphine that inhibited bacterial growth by 50% was evaluated using the plate count method. This concentration, called C_50_, was 0.27 ± 0.02 mM for the WT strain vs. 0.34 ± 0.01 mM for MG1655-*hfq72* (Figure 4). These values are the average of multiple experiments (see methods for details). The *E. coli* strain that does not express the CTR of Hfq (*hfq72*) is significantly less sensitive to apomorphine than the WT strain. C_50_ for the strain devoid of Hfq (MG1655-∆*hfq*) was 0.18 ± 0.02 mM (Figure 4). This result was expected since Hfq (with sRNAs) establishes resistance to various antibiotics [36]. A similar effect was observed for EGCG [28]. All p-values (for all pairwise comparisons) were smaller than 2 × 10^−5^ and indicated that all three strains had significantly different medians.

## 3. Discussion

Amyloid inhibitors, such as the green tea compound Epigallocatechin gallate EGCG or curlicide, have antibacterial properties [28,29,37]. Conversely, antibiotics such as tetracycline derivatives or rifampicin also affect eukaryotic amyloids’ formation [38,39] and may be used to treat neurodegenerative diseases. This opens the possibility for existing drugs to be repurposed in view of new therapy, targeting amyloid-like proteins from eukaryotes to prokaryotes and vice versa.

Here, we show that a different amyloid inhibitor than EGCG is also able to block bacterial development by targeting the Hfq riboregulator, and more precisely the *E. coli* Hfq C-terminal region. In this work, we characterize the effect of apomorphine, a dopamine agonist, on Hfq-CTR amyloid self-assembly in vitro and on bacterial survival and show that apomorphine, which is commonly used to treat Parkinson’s, emerges as a promising new antibacterial agent against Gram-negative bacteria. We observed that apomorphine significantly affected bacterial survival at a concentration of 1 mM. Taking into consideration that apomorphine delivered in patients treated for Parkinson’s disease is accompanied by a C_max_ of approximately 20 ng/mL (i.e., in the few hundreds of micromolars range), that it is well tolerated [40,41], and that new formulations have allowed a 10-fold increase in apomorphine bioavailability (due, for instance, to solid lipid nanoparticles) [31], it is noteworthy that the concentration of apomorphine allowing for antibacterial effects and that attained with good tolerance in patients are rather close. Therefore, the accurate determination of the apomorphine concentration needed in vivo in order to affect bacterial survival remains to be determined and compared to the tolerated doses of apomorphine, although we think that apomorphine could achieve these requirements so as to repurpose apomorphine as antibiotics. It should also be noted that another strategy would be that apomorphine could be considered as a lead compound scaffold for a new class of antibiotics that could serve for optimization in order to improve its pharmacokinetic and/or pharmacodynamic properties (see below).

Note that Aβ peptide (involved in Alzheimer’s disease) has also been recently recognized as an antimicrobial peptide [42]: Aβ oligomers have antimicrobial properties by forming fibrils that entrap bacteria and disrupt their cell membranes [43]. We thus need to be careful with the use of anti-amyloid compounds and be attentive that they do not affect the integrity of this natural protection of eucaryotic cells against bacteria.

The efficiency of apomorphine may thus need optimization in order to be used as an antibiotic in the future. In particular, it may need synthetic chemistry to more specifically target the Hfq-CTR amyloid region and not the host’s amyloid-like structures with beneficial properties. We know that the minimal amyloid region of Hfq-CTR consists in 11 amino acid residues within the 38 amino acid sequence of the CTR (underlined: SRPVSHHSNNAGGGTSSNYHHGSSAQNTSAQQDSEETE) [23]. Mechanistically, the autoxidation of apomorphine [44] is known to produce an unstable o-quinone form, which forms an adduct with nucleophilic groups of amyloids in order to inhibit their formation [45]. Thus, in the case of Hfq it is probable that the histidines, aspartic acid, glutamic acids and tyrosine residues close to the 11 amino acid sequence in Hfq-CTR contribute to apomorphine binding. The modification of the compound in order to allow it to react efficiently and specifically with Hfq-CTR will be necessary. Nevertheless, the precise atomic 3D structure of this region of the protein is still unknown [7]. Many crystallization tentatives, solid state NMR or molecular modelling approaches have been used over twenty years [16,46,47], but as of now they have all failed. A rational design to find a more efficient modified apomorphine to target Hfq-CTR is thus a challenge. As many properties of this protein depend on its CTR (membrane remodeling, DNA compaction, sRNAs recycling… [23,32,48]), efforts to obtain a structure for this region should be made to provide new clues in order to target important functions of this bacterial master regulator and affect bacterial survival. Considering the recent advance in cryo-TEM that enables a macromolecular structure determination with and without antibiotics in vitro and now even in vivo [49], one possibility would be to try to determine the structure of Hfq amyloid fibers using high-resolution cryo-TEM with and without apomorphine [49]. Furthermore, it is possible that the concentration needed in vivo to affect bacterial survival will be higher than that used in vitro in our analyses. This also justifies a probable optimization of the compound for it to be effective.

## 4. Materials and Methods

### 4.1. Chemicals

All chemicals, including apomorphine (IUPAC name: (6aR)-6-methyl-5,6,6a,7-tetrahydro-4H-dibenzo[de,g]quinoline-10,11-diol;hydrochloride), were purchased from Sigma-Aldrich. Apomorphine was prepared in milliQ water at 10 mM.

### 4.2. Hfq CTR Peptides

Hfq-CTR peptide (SRPVSHHSNNAGGGTSSNYHHGSSAQNTSAQQDSEETE) was chemically synthetized and prepared as described previously in Partouche et al. [28]. DNA-induced fibrillation was described previously in [32,50,51]. As salts, pH and temperature may influence the stability of amyloid self-assembly [52], we first check if our fibers are stable in our experimental conditions. To this end, we used SRCD and followed the decrease in the amyloid signal at 215 nm. We evaluated that the melting temperature of the Hfq-CTR amyloid structure was about 80 °C in our experimental conditions. Note that adding salts results in noise in deep-UV SRCD measurements and is thus avoided for our analysis.

### 4.3. TEM Imaging of Protein Fibrils

Apomorphine was added to fibrils (final concentrations from 0.5 to 5 mM). Except when specified, samples were incubated for 24 h and visualized by TEM. To perform negative staining, 5 μL of the peptide sample was deposited on a glow-discharged electron microscopy 200 mesh copper grid coated with a continuous film of carbon (purchased from EMS). After 2 min of interaction with the grid, the excess sample was removed using Whatman filter paper. Then, 5 μL of contrasting agent solution (Gadolinium salt, uranyl-less) was applied onto the grid with peptide. After a 1-min incubation time, the excess of contrasting agent was blotted out with Whatman filter paper, and then the grids were kept in a grid box (a dry, dark and dust-free environment) until observation with the electron microscope. The grids were mounted onto a room temperature holder and were introduced into and observed with a JEOL 2200FS electron microscope (JEOL, Tokyo, Japan). The TEM images (2048 × 2048 pixels) were acquired with a Gatan US1000 slow scan CCD camera at various magnifications depending on the type of experiments. The displayed images are representative of what was observed on the whole grids.

### 4.4. Synchrotron Radiation Circular Dichroism (SRCD)

SRCD analysis measurements were carried out on DISCO beamline at the SOLEIL Synchrotron as described previously (proposal #20180165) [53]. 4 µL of samples were loaded into circular demountable CaF_2_ cells with a 4.7-micron path length [54]. Data analyses were carried out with CDtool [55]. The spectral cut-off was set to 175 nm according to the high tension midpoint at 410 V of the photomultiplier. The secondary structure content was determined using BestSel [33,34]. Disassembly of the Hfq-CTR fibers structure by apomorphine at 5 mM was recorded for 15 h. The decrease in the CD signal at 215 nm, which is characteristic of the amyloid structure, was reported as a function of time. In our case, the apomorphine concentration was significantly higher than fibers (referred to as [Hfq-CTR_n_]), and the absolute value of the CD signal at 215 nm CD_215_ = CD^0^_215_ × e^−kt^. CD^0^_215_ is the CD signal at 215 nm at t = 0, and k is the apparent dissociation constant of the reaction.

### 4.5. Construction of E. coli Strains

Strains were constructed with the λ-red recombination technique, as described in Malabirade et al. [32].

### 4.6. Effect of Apomorphine on E. coli Survival

The effect of apomorphine on bacterial survival was evaluated using the plate count method, as described previously in Partouche et al. [28]. Briefly, apomorphine ranging from 0 to 0.5 mM was incorporated into an LB agar medium, and a standardized number of cells (three dilutions from 10^−5^ to 10^−7^ cells/mL) were plated in quadruplicate, followed by overnight incubation at 37 °C [56]. The experiment was repeated with at least three independent cultures to ensure the statistical significance of the analysis.

### 4.7. Statistical Analysis

As some data did not follow a normal distribution, a Kruskal–Wallis test was performed and showed (*p* value < 2 × 10^−7^) that at least one sample stochastically dominated another sample. Subsequently, multiple Wilcoxon rank-sum tests were performed and were adjusted to control Type I error rates. Here, all p-values (for all pairwise comparisons) were smaller than 2 × 10^−5^ and indicated that all three strains had different medians. All analyses were performed with R (https://www.R-project.org/) (4 December 2020) [57].

## 5. Conclusions

The role of functional amyloids as bacterial virulence factors is diverse, even if it can sometimes be indirect [58]. Here, we show that they can be promising targets to develop new antibiotics. More precisely, apomorphine, used in the treatment of Parkinson’s, can be used for this goal. If apomorphine has a low effect when taken orally (due to a fast liver metabolism), it can be given subcutaneously at a concentration compatible with that used in order to affect bacterial survival in our present analysis [31]. It could thus be a promising drug to treat local infections such as skin or surgical site infections caused by Gram-negative bacteria. Furthermore, progress in drug delivery could help in obtaining a broader use of this compound or its derivative in the near future [31].

## Data Availability

The data presented in this study are available on request to the corresponding.
